# Supplementary material for: Toward universal cell embeddings: integrating single-cell RNA-seq datasets across species with SATURN
Source: Nat Methods. 2024 Feb 16;21(8):1492–500. doi: 10.1038/s41592-024-02191-z (PMC11310084; doi:10.1038/s41592-024-02191-z)
Supplement: Supplementary file 1 — Supplementary Figs. 1–5, Notes 1–6, Tables 1–3 and References. [file 41592_2024_2191_MOESM1_ESM.pdf]

nature methods

Article https://doi.org/10.1038/s41592-024-02191-z

Toward universal cell embeddings: integrating single-cell RNA-seq datasets across species with SATURN

In the format provided by the authors and unedited

Supplementary materials for

Towards Universal Cell Embeddings: Integrating Single-cell RNA-seq Datasets across Species with SATURN

Yanay Rosen[1,*] Maria Brbić[2,*], Yusuf Roohani[3,*], Kyle Swanson[1], Ziang Li[4], Jure Leskovec[1,†]

[1] Department of Computer Science, Stanford University, Stanford, CA, USA

[2] School of Computer and Communication Sciences, Swiss Federal Institute of Technology (EPFL), Lausanne, Switzerland

[3] Department of Biomedical Data Science, Stanford University, Stanford, CA, USA

[4] Department of Computer Science and Technology, Tsinghua University, Beijing, China

[†]Corresponding author. Email: jure@cs.stanford.edu

[*]These authors contributed equally

This PDF file includes:

Supplementary Notes 1 to 6
Supplementary Figures 1 to 5
Supplementary Tables 1 to 3
Supplementary References

Supplementary Note 1 Datasets and preprocessing

We downloaded publicly available count matrix files with cell type annotations (see data availability). For integrating Tabula Sapiens [1], Tabula Microcebus [2] and Tabula Muris [3] we filtered cell types to select cell types with more than 350 cells. Additionally, we filtered cells with fewer than 500 genes expressed and filtered genes expressed in fewer than 1000 cells. For frog and zebrafish embryogenesis, we filtered cells with fewer than 500 genes expressed, and filtered genes that were expressed in fewer than 10 cells. For the Aqueous Humor Outflow cell atlas no additional gene or cell filtering was done. We selected highly variable genes in each dataset using the Seurat v3 method [4]. We set only number of genes (Supplementary Note 4), while we keep all other parameters to their default values in scanpy package [5]. No additional data preprocessing was performed and the numerical inputs to SATURN are raw counts.

Supplementary Note 2 Baseline methods

We compare SATURN to four existing single cell integration methods, SAMap, Harmony, scVI and Scanorama. SAMap is run in a semi-supervised mode in which cell neighborhoods are determined by cell types. Harmony [6], scVI [7], and Scanorama [8] are all run with default settings, using one-to-one homolog genes, and with the batch variable being species (frog or zebrafish). For scVI, Harmony and Scanorama, no additional highly variable gene selection was performed as the number of one-to-one homologs was low (7175). SAMap defaults to 3000 highly variable genes for each species, as determined by their SAM weights.

Supplementary Note 3 Evaluation

There are a variety of different ways to assess the quality of a multi-species embedding. A multi-species embedding should encode cells that are the same cell type close together and cells from different cell types far apart. Cell types that are shared across species should have similar embeddings, and cell types that are unique to a species should not be falsely paired with other cell types.

We therefore assess the quality of multi-species embeddings for the goal of transferring labels from one species to another. Given a species s^1 with distinct cell types T^1, labels are transferred to a new species s^2 with cell types T^2 using a cell type classifier trained on the embeddings of cells from s^1. The simple classification model $C_{s^1}(\mathbf{z}_c) : \mathbb{R} \rightarrow T^1$ is trained on embeddings of one species s^1, and evaluated on embeddings of another species s^2. Predictions are classified as accurate based on a predetermined mapping of cell types $T^1 \rightarrow T^2$ between species (Supplementary Table 2).

$$C_{s^1} := \text{Logistic Regression Model}(\mathbf{z}_{c \in s^1}) \sim T^1_{c \in s^1} \tag{1}$$

$$\hat{T}^1_{c \in s^2} = C_{s^1}(\mathbf{z}_{c \in s^2}) \tag{2}$$

$$\text{Accuracy} = \frac{1}{|c \in s^2|} \sum_{c \in s^2} \mathbb{1}(\hat{T}^1_c \text{ maps to } T^2_c) \tag{3}$$

Supplementary Note 4 Hyperparameters

Hyperparameters. In SATURN, we set the number of highly variable genes to 8000. For integrating frog and zebrafish embryogenesis datasets and integrating the AH atlas, the number of macrogenes $|\mathcal{M}|$ is 2000. For integrating tissue subsets of the mammalian atlas datasets, the number of macrogenes is 3000. This dataset requires integration of fine-grained cell types from closely related species so we set the number of macrogenes to higher value. Intuitively, a higher number of macrogenes may help in finding finer-level differences between cell types, as an increased number of macrogenes will result in a more specific gene grouping. However, increasing the number of macrogenes past a certain point could reduce interpretability as the macrogenes may become too specific and consist of single genes. Since we are reducing the original high-dimensional gene space from all species in the macrogene space, we do not recommend using fewer than 1000 macrogenes. The encoder embedding dimension, k is set at 256 for all experiments. The hidden dimension for all other layers used during pretraining is 256. We use Adam optimizer with learning rate 0.0005 during pretraining and 0.001 during fine-tuning with metric learning.

To generate the coarse alignment of mammalian cell atlases in Fig. 1b, SATURN was run with 8000 highly variable genes per species, 2000 macrogenes, an embedding dimension k of 256 and a hidden dimension of 256. An additional categorical covariate was added to the embedding dimension, representing the tissue of origin. All UMAP visualizations are generated using default values in scanpy package [5]. We generate UMAP embeddings with randomized plotting order in Supplementary Fig. 1.

Supplementary Note 5   Gene Ontology enrichment analysis

Gene Ontology (GO) analysis could additionally confirm functionally meaningful groups of macrogenes. However, the challenge is that many species do not have well annotated GO terms and mapping GO terms across different species is non-trivial. Thus, we performed GO term enrichment analysis between human and mouse in the mammalian cell atlas, since human and mouse genes are best annotated in the GO. To create gene sets, for each macrogene we took the set of a given species' (either mouse or human) genes that had weights from a gene to macrogene above a cutoff of 0.5. From these, to ensure gene sets had a sufficient size for enrichment analysis, we selected gene sets with 10 or more genes, and ran GO enrichment analysis using the GOATOOLS Python package [9].

Using this approach, 88 human gene sets and 79 mouse gene sets were created. GO enrichment analysis on the human gene sets found an average of 2.05 biological process (BP) terms, 1.35 molecular function (MF) terms and 1.88 cellular component (CC) terms that were enriched at a significant level (p=0.05, FDR BH corrected, default parameters) per human gene set. Enrichment analysis on the mouse gene sets found an average of 4.10 BP terms, 2.38 MF terms and 2.86 CC significant terms per mouse gene set. In the null distribution of random assignment of genes, 0 sets had significant terms of any kind. Moreover, we found 14 macrogenes for which we could create gene sets for both human and mouse. In $11/14$ of these macrogenes we found at least one significantly enriched GO term in common between the mouse and human sets when performing string-based matching of terms.

Supplementary Note 6 Macrogene initialization functions

Default initialization. By default, SATURN initializes macrogenes by soft-clustering protein embeddings. In particular, SATURN first clusters protein embeddings using the K-Means algorithm [10]. Given a matrix that stores protein embeddings for all genes $\mathbf{P} \in \mathbb{R}^{|\mathcal{G}| \times p}$, SATURN applies K-Means to learn a set of centroids $\mathcal{M} = \{\mathbf{m}_i \in \mathbb{R}^p\}_{i=1}^{N_M}$ where N_M defines the number of centroids/macrogenes. K-means minimizes the within-cluster sum of squares:

$$\sum_{g \in \mathcal{G}} min_{\mathbf{m} \in \mathcal{M}}(||\mathbf{P}_g - \mathbf{m}||^2), \tag{4}$$

where $\mathbf{P}_g$ denotes a row protein embedding vector of matrix $\mathbf{P}$. Here, each centroid $\mathbf{m}$ represents a different macrogene. SATURN then defines an initial set of weights $\{\{\mathbf{W}_{g,m} \in \mathbb{R}+\}_{g=1}^{|\mathcal{G}|}\}_{m=1}^{|\mathcal{M}|}$ from each gene g to each macrogene m as:

$$\mathbf{W}_{g,m} = 2 * \left(\log\left(\frac{1}{\text{rd}_{m,g}} + 1\right)\right)^2, \tag{5}$$

where $\text{rd}_{m,g} : \mathbb{N} \rightarrow \mathbb{N}$ represents the ranked euclidean distance from gene g to a macrogene m and $\text{rd}_{m,g} = 1$ for the nearest gene to a macrogene. This initialization function is arbitrarily chosen so that genes have the highest weights to the macrogenes they are closest to. Gene to macrogene weights are strictly positive, differentiable and updated during pretraining. We multiply by two so that the highest weights are close to 1.

Additional Functions. We benchmark two additional initialization functions, a smoother function and an all-or-nothing "one-hot" function, which perform similarly (Supplementary Fig. 4).

For the more smoothed initialization function, the weights $\{\{\mathbf{W}_{g,m} \in \mathbb{R}+\}_{g=1}^{|\mathcal{G}|}\}_{m=1}^{|\mathcal{M}|}$ from each gene g to each macrogene m are set as:

$$\mathbf{W}_{g,m} = \frac{1}{\text{rd}_{m,g}} \tag{6}$$

For the one hot initialization function, the weights $\{\{\mathbf{W}_{g,m} \in \mathbb{R}+\}_{g=1}^{|\mathcal{G}|}\}_{m=1}^{|\mathcal{M}|}$ from each gene g to each macrogene m are set as:

$$\mathbf{W}_{g,m} = \mathbb{1}(\text{rd}_{m,g} = 1) \tag{7}$$

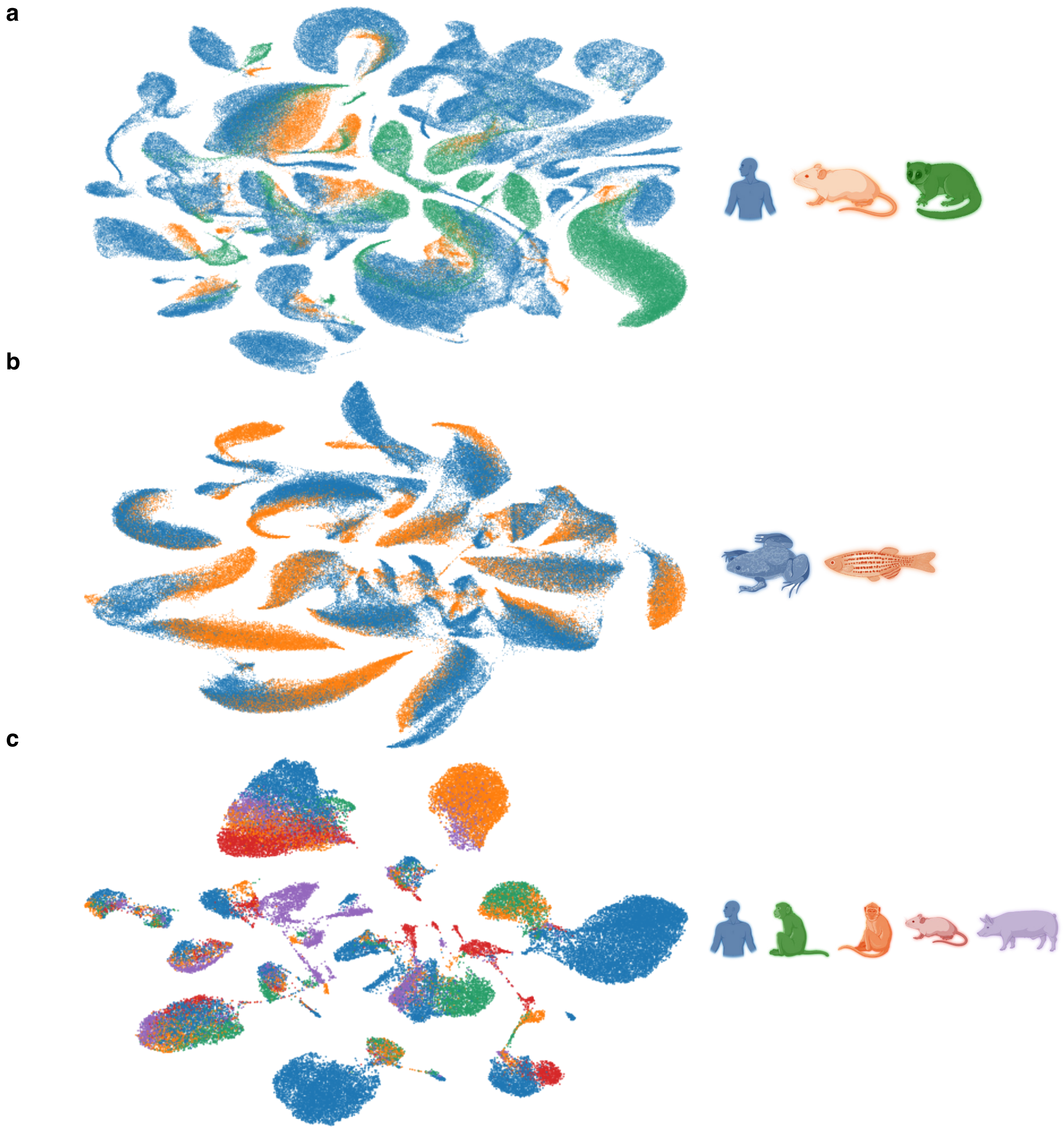

Supplementary Figure 1: SATURN embeds multi species datasets. UMAP embeddings of **(a)** mammalian cell atlas, **(b)** frog and zebrafish embryogenesis datasets and **(c)** Aqueous Humor Outflow cell atlas. UMAPs are generated using default parameters but plotting order is randomized.

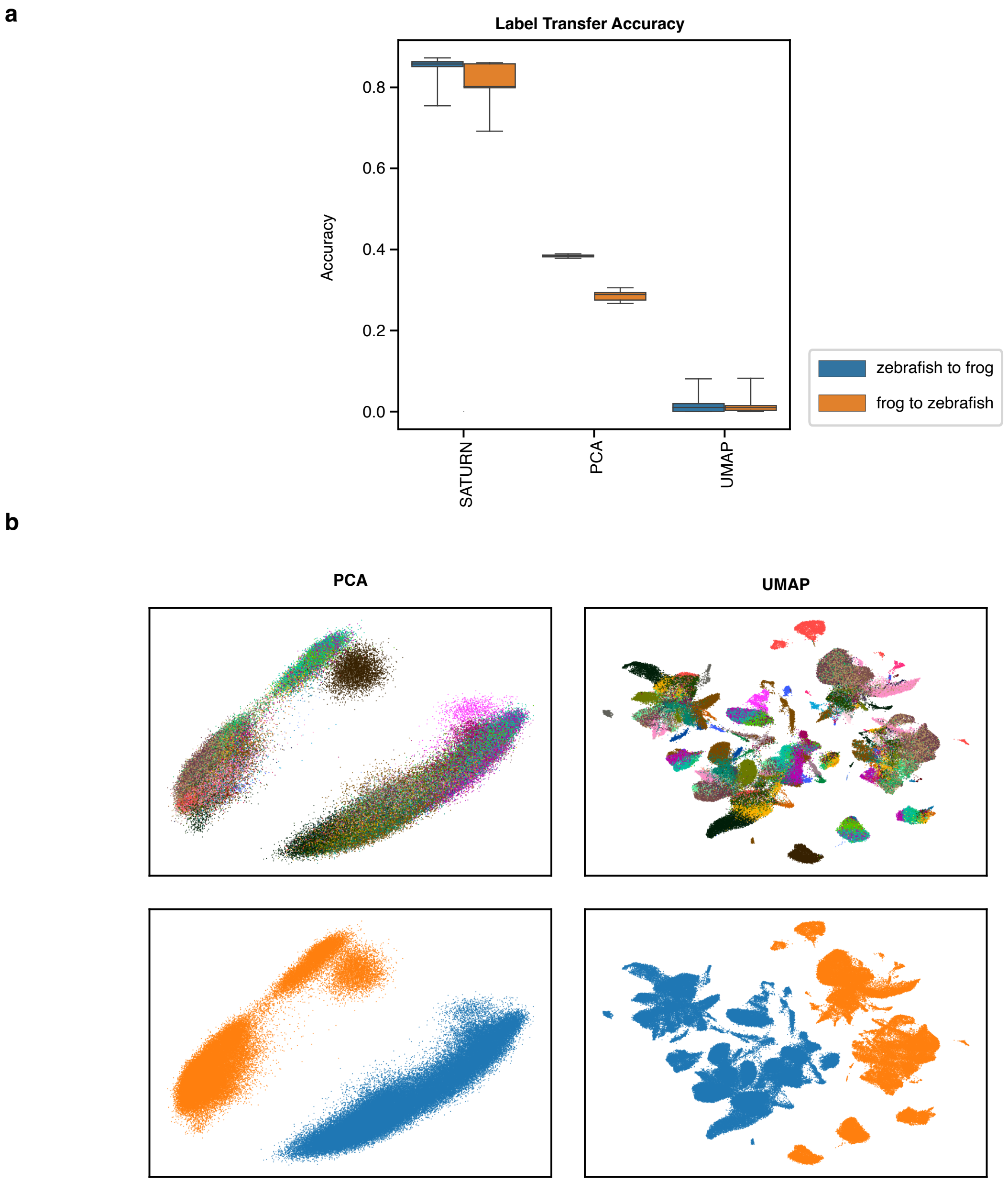

Supplementary Figure 2: SATURN outperforms UMAP and PCA for cross species integration. **(a)** Performance comparison of SATURN versus PCA and UMAP on frog and zebrafish embryogenesis datasets. PCA is calculated using the one-to-one homolog genes as determined by BLAST, followed by expression log normalization. UMAP is then calculated using those top 50 principal components. The distribution is obtained with n=30 runs for each method, by setting a random seed and shuffling the data. **B** Visualization of PCA (left) and UMAP (right) embeddings by cell type (top) and species (bottom). For PCA, the top two principal components are used.

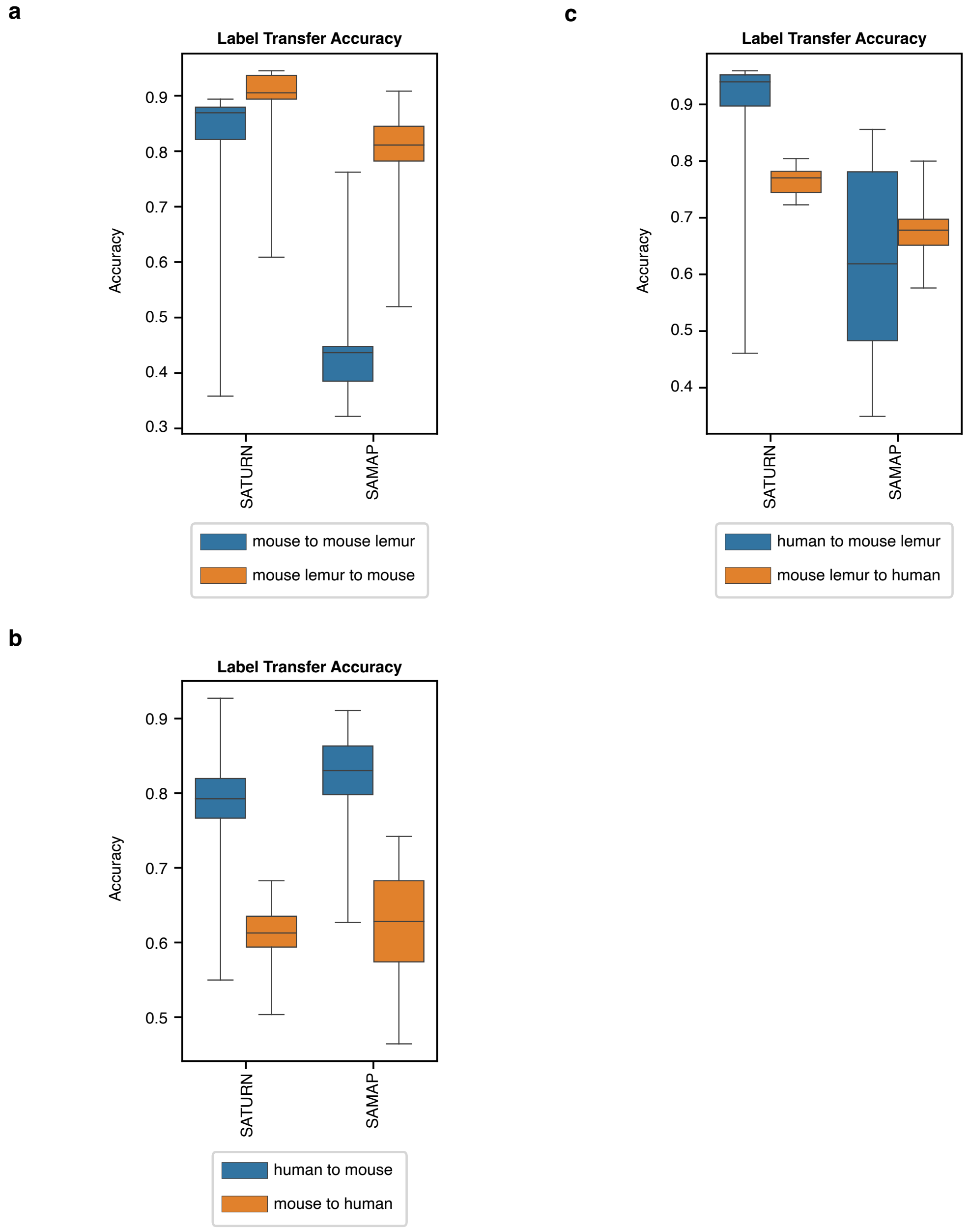

Supplementary Figure 3: Performance of SATURN and the second best baseline SAMap on transferring annotations on the mammalian cell atlas. Performance is evaluated using the prediction accuracy of a logistic classifier model trained to differentiate cell types of one species and tested on predicting the cell type annotations of another species. Higher values indicate better performance. SAMap represents a version of the SAMap method in which cell-type annotations are used to integrate datasets. The distribution is obtained with n=30 runs for each method. Performance when transferring annotations from **(a)** mouse to mouse lemur, **(b)** human to mouse, and **(c)** human to mouse lemur.

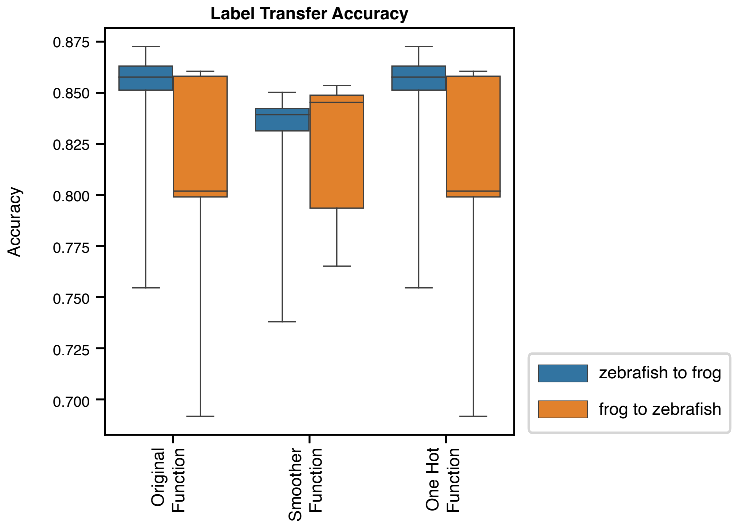

Supplementary Figure 4: SATURN is robust to choice of macrogene initialization function. Median performance of SATURN with different macrogene initialization functions evaluated as accuracy of the label transfer between frog and zebrafish embryogenesis datasets. Blue boxplots show zebrafish to frog label transfer performance, while orange boxplots show frog to zebrafish label transfer performance. Distribution is estimated with n = 30 runs.

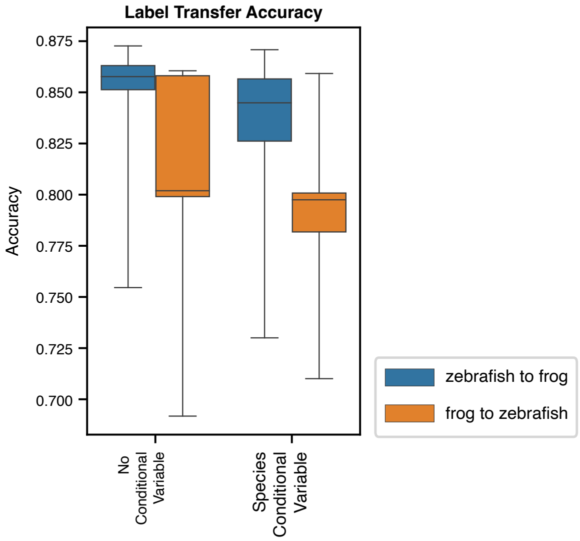

Supplementary Figure 5: Conditional species variable does not improve performance. Performance of SATURN using a conditional autoencoder during pretraining with a species conditional variable vs a constant variable. The constant variable is appended to the embedding $\mathbf{z}_c$, while in the conditional variable setting, a one hot representation of the species s is concatenated to the embedding. Blue boxplots show zebrafish to frog label transfer performance, while orange boxplots show frog to zebrafish label transfer performance. Distribution is estimated with $n = 30$ runs.

**Macrophage and Myleoid Progenitors**									
**Arhgdi**		**Cebp**		**Ptp**		**Cybb**		**Lcp1**	
**gene**	**weight**	**gene**	**weight**	**gene**	**weight**	**gene**	**weight**	**gene**	**weight**
frog_arhgdib	2.0586648	frog_cebpd	1.7377852	frog_ptprc	1.0184758	frog_cybb	1.4121561	frog_lcp1	1.1437993
zebrafish_arhgdig	1.1892534	frog_cebpb	1.2713358	zebrafish_ptprc	1.0062823	zebrafish_cybb	0.98110956	zebrafish_parvg	0.95570034
frog_arhgdig	0.82862365	zebrafish_cebpa	1.2589424	frog_iqcd	1.0023459	frog_nox4	0.69433695	zebrafish_parvb	0.8877349
frog_arhgdia	0.42018056	zebrafish_cebpb	1.1590556	frog_ptpn6	0.975968	frog_nox1	0.6060228	frog_parva	0.8088149
frog_c20orf27	0.012586672	frog_mafb	0.777314	zebrafish_ptpreb	0.95713043	frog_nox5	0.016699424	zebrafish_lcp1	0.7965079
frog_arr3	0.011929505	zebrafish_cebpd	0.6585815	zebrafish_ptpn6	0.9493796	frog_nadk	0.012460865	frog_parvb	0.7859018
zebrafish_abracl	0.0018043627	frog_cebpa	0.49666032	zebrafish_ptpn22	0.88780427	zebrafish_slc7a8a	0.008461936	frog_parvg	0.6744168
zebrafish_c7h20orf27	0.0017385085	zebrafish_mafbb	0.3763802	zebrafish_ptpn11b	0.5531652	frog_rac2	0.006649947	zebrafish_parvab	0.62013125
zebrafish_arr3b	0.0011982815	zebrafish_cebp1	0.2561873	zebrafish_ptprr	0.533596	zebrafish_slc7a7	0.006204006	zebrafish_gas2l2	0.2786125
zebrafish_cst14b.1	0.0007732745	frog_maf	0.2419157	frog_ptprh	0.39319068	frog_tfb1m	0.006183132	zebrafish_tagln	0.11108811
**Ionocytes**									
**Foxi**		**Dmrt2**		**Cldn**		**Ubp1**		**Atp60v**	
**gene**	**weight**	**gene**	**weight**	**gene**	**weight**	**gene**	**weight**	**gene**	**weight**
frog_foxi1	1.7946975	frog_dmrt2	1.1082501	zebrafish_cldna	0.47487783	frog_ubp1	1.4286531	frog_atp6v0c	1.5118276
zebrafish_foxi3a	1.7876347	zebrafish_dmrt2a	1.0135943	zebrafish_cldnh	0.46664542	frog_grhl3	1.1210046	zebrafish_atp6v0cb	1.0511838
zebrafish_foxi1	1.7752392	frog_kank1	0.9281069	frog_cldn4	0.45497242	frog_grhl1	1.0977421	zebrafish_atp6v0ca	0.6842436
zebrafish_foxg1a	1.767095	zebrafish_gcm2	0.4140955	zebrafish_cldnb	0.40522912	zebrafish_grhl3	1.03748	frog_atp6v0b	0.33991408
frog_foxg1	1.6482608	zebrafish_dmrt2b	0.3073387	zebrafish_cldne	0.38158783	zebrafish_grhl2a	0.85601187	zebrafish_atp6v0b	0.18918027
frog_foxi4.2	1.6366866	zebrafish_cxxc4	0.14239863	zebrafish_lhfpl3	0.30014035	zebrafish_tp63	0.6001469	frog_cnih1	0.0017733343
frog_foxi2	1.598392	frog_cxxc4	0.1175361	zebrafish_cldnc	0.24535778	frog_grhl2	0.54704624	frog_sec61g	0.0010816682
zebrafish_foxg1b	0.92830807	frog_foxi1	0.09924057	frog_lhfpl4	0.24350967	zebrafish_grhl1	0.44004646	frog_eif1ax	0.00058003364
zebrafish_foxh1	0.7387778	zebrafish_skor1b	0.09714537	zebrafish_lhfpl5a	0.2244674	zebrafish_tfcp2l1	0.4264244	zebrafish_sec61g	0.00044005408
frog_foxe1	0.48347607	frog_hivep1	0.07280374	zebrafish_lhfpl5b	0.18170683	frog_tp63	0.30589458	zebrafish_rpl34	0.0004284728

Supplementary Table 1: Frog and Zebrafish differentially expressed macrogenes' gene to macrogene weights. Gene to macrogene weights for the top 10 genes for each differentially expressed macrogene in Figure 2b. Genes are listed in descending order by weight.

Frog Cell Type	Zebrafish Cell Type	# of Frog Cells	# of Zebrafish Cells	Total # of Cells
Hindbrain	Hindbrain	7273	9399	16672
Intermediate mesoderm	Intermediate mesoderm	10324	3120	13444
Forebrain/midbrain	Forebrain/midbrain	2081	10500	12581
Epidermal progenitor	Epidermal progenitor	9149	1921	11070
Non-neural ectoderm	Non-neural ectoderm	8022	2227	10249
Neural crest	Neural crest	8393	1769	10162
Neuroectoderm	Neuroectoderm	6590	3381	9971
Placodal area	Placodal area	6918	1188	8106
Presomitic mesoderm	Presomitic mesoderm	6293	1642	7935
Skeletal muscle	Skeletal muscle	5772	651	6423
Neuron	Neuron	1899	4032	5931
Tailbud	Tailbud	1860	3759	5619
Optic	Optic	1475	3676	5151
Blood	Blood	1569	3067	4636
	Pluripotent	0	4277	4277
Involuting marginal zone	Involuting marginal zone	2385	1849	4234
Endoderm	Endoderm	2207	890	3097
Eye primordium	Eye primordium	2477	223	2700
Endothelial	Endothelial	1002	884	1886
Goblet cell		1473	0	1473
Small secretory cells		1335	0	1335
Ionocyte	Ionocyte	1030	292	1322
Notochord	Notochord	766	351	1117
Blastula		1116	0	1116
Otic placode	Otic placode	813	270	1083
Heart	Heart	121	851	972
Spemann organizer		963	0	963
Myeloid progenitors		778	0	778
Pronephric mesenchyme		777	0	777
Cement gland primordium		721	0	721
Lens	Lens	458	210	668
	Rare epidermal subtypes	0	513	513
Notoplate	Notoplate	339	115	454
Rohon-beard neuron	Rohon-beard neuron	134	289	423
Olfactory placode	Olfactory placode	139	276	415
	Macrophage	0	405	405
	Periderm	0	382	382
Hatching gland	Hatching gland	180	82	262
	Dorsal organizer	0	233	233
	Pharyngeal pouch	0	209	209
	Apoptotic-like	0	163	163
	Pronephric duct	0	95	95
Germline	Germline	33	53	86
Neuroendocrine cell		70	0	70
	Pancreas primordium	0	49	49
	Secretory epidermal	0	34	34
	Apoptotic-like 2	0	33	33
	Forerunner cells	0	5	5
	Epiphysis	0	3	3
	Nanog-high	0	3	3
Totals: 36	42	96935	63371	160306

Supplementary Table 2: Cell Type Matching and Frequencies in Frog and Zebrafish Embryogenesis. Cell type pairs used for scoring frog and zebrafish embryogenesis embeddings, and cell type counts.

Cluster	Macrogene	Human Genes	Cynomologus Macaque Genes	Rhesus Macaque Genes	Mouse Genes	Pig Genes
1	1540	Col6A2, Vit, Col6A6	Vit, Col28A1, Antxr2	Col6A2, Col28A1, Vit	Col6A1, Col6A2, Vit	Vit, Antxr2, Col6A2
1	71	Rpp25, Sco2, Siglec1	Adam15, Siglec1, Nop9	Adam15, Lhb, Kcp	Ptpn18	C4A, Kcnk7, Rpp25
2	1115	Cxcl12, Ccl25	Cxcl12	Ccl25, Cxcl14	Cxcl12	Cxcl12
2	197	Nr2F1, Nr2F2	Nr2F1, Nr2E3, Nr2E1	Nr2F2, Nr2F1, Nr2E3	Nr2F1, Nr2F2, Nr2E3	Nr2F1, Nr2F2, Nr0B2
3	232	Tagln, Tagln2, Tagln3	Tagln, Tagln3	Tagln, Tagln2	Tagln, Tagln3	Tagln, Tagln3
3	583	Rspo2, Rspo3	Rspo2, Rspo3	Rspo2, Rspo3	Rspo3, Rspo2, Rspo1	Rspo3, Rspo2
4	748	Bgn				
4	433	Prelp, Ogn, Aspn	Ogn, Kera, Prelp	Ogn, Prelp, Optc	Dcn, Fmod, Optc	Omd, Ogn, Ecm2
4	479	Angptl7, Fgl2, Angptl1	Fgl2, Angptl7, Fgb	Fgl2, Angptl7, Fgg	Fgl2, Angptl7, Angptl2	Fgl2, Angptl7, Fibcd1
4	1273	Tnxb, Matn2, Tnr	Tnc, Morn4	Morn4, Tnc, Matn2	Tnxb	Zcchc13, Tnc, Tnr
5	1300	Ca3, Ca13, Ca7	Ca2, Ca7, Ca3	Ca3, Ca2, Ca13	Car3, Car2, Car13	Ca2, Ca3, Ca7
5	73	Slc4A7, Slc4A4, Slc4A10	Slc4A10, Slc4A7, Slc4A4	Slc4A10, Slc4A7, Slc4A4	Slc4A4, Slc4A5	Slc4A4, Slc4A7, Slc4A5
5	97	Fgf6, Fgf23, Fgf16	Fgf21, Fgf19, Fgf10	Fgf10, Fgf8, Fgf9	Fgf10, Fgf5, Fgf21	Fgf10, Fgf22, Fgf21

Supplementary Table 3: Differentially expressed macrogenes in regrouped AH Atlas cell types. Genes in the table represent the corresponding species' top 3 genes per macrogene, ordered by weight and with weights above 0.5.

Supplementary Data References

1. Tabula Sapiens Consortium et al. The Tabula Sapiens: A multiple-organ, single-cell transcriptomic atlas of humans. *Science* **376**, eabl4896 (2022).

2. Consortium, T. T. M. *et al.* Tabula Microcebus: A transcriptomic cell atlas of mouse lemur, an emerging primate model organism. *BioRxiv* (2021).

3. Tabula Muris Consortium. Single-cell transcriptomics of 20 mouse organs creates a Tabula Muris. *Nature* **562**, 367–372 (2018).

4. Butler, A., Hoffman, P., Smibert, P., Papalexi, E. & Satija, R. Integrating single-cell transcriptomic data across different conditions, technologies, and species. *Nature Biotechnology* **36**, 411–420 (2018).

5. Wolf, F. A., Angerer, P. & Theis, F. J. SCANPY: large-scale single-cell gene expression data analysis. *Genome Biology* **19**, 15 (2018).

6. Korsunsky, I. *et al.* Fast, sensitive and accurate integration of single-cell data with Harmony. *Nature Methods* **16**, 1289–1296 (2019).

7. Lopez, R., Regier, J., Cole, M. B., Jordan, M. I. & Yosef, N. Deep generative modeling for single-cell transcriptomics. *Nature Methods* **15**, 1053–1058 (2018).

8. Hie, B., Bryson, B. & Berger, B. Efficient integration of heterogeneous single-cell transcriptomes using Scanorama. *Nature Biotechnology* **37**, 685–691 (2019).

9. Klopfenstein, D. *et al.* GOATOOLS: A python library for gene ontology analyses. *Scientific reports* **8**, 1–17 (2018).

10. Lloyd, S. Least squares quantization in PCM. *IEEE Transactions on Information Theory* **28**, 129–137 (1982).
