# Supplementary material for: Toward universal cell embeddings: integrating single-cell RNA-seq datasets across species with SATURN
Source: Nat Methods. 2024 Feb 16;21(8):1492–500. doi: 10.1038/s41592-024-02191-z (PMC11310084; doi:10.1038/s41592-024-02191-z)
Supplement: Supplementary file 2 — Reporting Summary [file 41592_2024_2191_MOESM2_ESM.pdf]

nature portfolio

Corresponding author(s): Jure Leskovec

Last updated by author(s): 2023/12/05

Reporting Summary

Nature Portfolio wishes to improve the reproducibility of the work that we publish. This form provides structure for consistency and transparency in reporting. For further information on Nature Portfolio policies, see our Editorial Policies and the Editorial Policy Checklist.

Statistics

For all statistical analyses, confirm that the following items are present in the figure legend, table legend, main text, or Methods section.

n/a	Confirmed	
☐	☒	The exact sample size (*n*) for each experimental group/condition, given as a discrete number and unit of measurement
☐	☒	A statement on whether measurements were taken from distinct samples or whether the same sample was measured repeatedly
☐	☒	The statistical test(s) used AND whether they are one- or two-sided<br>*Only common tests should be described solely by name; describe more complex techniques in the Methods section.*
☒	☐	A description of all covariates tested
☒	☐	A description of any assumptions or corrections, such as tests of normality and adjustment for multiple comparisons
☐	☒	A full description of the statistical parameters including central tendency (e.g. means) or other basic estimates (e.g. regression coefficient) AND variation (e.g. standard deviation) or associated estimates of uncertainty (e.g. confidence intervals)
☒	☐	For null hypothesis testing, the test statistic (e.g. *F*, *t*, *r*) with confidence intervals, effect sizes, degrees of freedom and *P* value noted<br>*Give P values as exact values whenever suitable.*
☒	☐	For Bayesian analysis, information on the choice of priors and Markov chain Monte Carlo settings
☒	☐	For hierarchical and complex designs, identification of the appropriate level for tests and full reporting of outcomes
☒	☐	Estimates of effect sizes (e.g. Cohen's *d*, Pearson's *r*), indicating how they were calculated

Our web collection on statistics for biologists contains articles on many of the points above.

Software and code

Policy information about availability of computer code

Data collection	We used code for proccessing scRNA-seq count data available here: https://github.com/snap-stanford/SATURN
Data analysis	SATURN was written in Python 3.8.1 using the PyTorch library (v1.13.1). The source code is available at https://github.com/snap-stanford/SATURN including code to download and process raw data. Additionally, SATURN outputs are available at http://snap.stanford.edu/saturn/data/. Homologs were determined using BLASTP version 2.9.0

For manuscripts utilizing custom algorithms or software that are central to the research but not yet described in published literature, software must be made available to editors and reviewers. We strongly encourage code deposition in a community repository (e.g. GitHub). See the Nature Portfolio guidelines for submitting code & software for further information.

Data

Policy information about availability of data

All manuscripts must include a data availability statement. This statement should provide the following information, where applicable:

- Accession codes, unique identifiers, or web links for publicly available datasets
- A description of any restrictions on data availability
- For clinical datasets or third party data, please ensure that the statement adheres to our policy

All analyzed datasets are publicly available and listed in the data availability section. Tabula Sapiens is available at CellXGene https://cellxgene.cziscience.com/collections/e5f58829-1a66-40b5-a624-9046778e74f5. Tabula Microcebus is available at FigShare https://figshare.com/articles/dataset/

Tabula_Microcebus_v1_0/14468196?file=31777475. Tabula Muris is available at FigShare https://figshare.com/articles/dataset/Single-cell_RNA-seq_data_from_microfluidic_emulsion_v2_/5968960/2. For embryogenesis datasets, frog is available with accession code GSE113074 and zebrafish is available in h5ad format at KleinTools. The five species aqueous humor outflow pathway atlas datasets are available with accession code GSE146188.

Human research participants

Policy information about studies involving human research participants and Sex and Gender in Research.

Reporting on sex and gender	N/A
Population characteristics	N/A
Recruitment	N/A
Ethics oversight	N/A

Note that full information on the approval of the study protocol must also be provided in the manuscript.

Field-specific reporting

Please select the one below that is the best fit for your research. If you are not sure, read the appropriate sections before making your selection.

☒ Life sciences ☐ Behavioural & social sciences ☐ Ecological, evolutionary & environmental sciences

For a reference copy of the document with all sections, see nature.com/documents/nr-reporting-summary-flat.pdf

Life sciences study design

All studies must disclose on these points even when the disclosure is negative.

Sample size	No samples were taken and the full dataset was used in each case for alignment.
Data exclusions	For aligning mammalian cell atlases, the cell atlases were subset to the list of common tissues in order to provide a more meaningful alignment.
Replication	Alignment benchmarks and reannotation results were verified by replicating SATURN results with 30 independent runs, each paramtetrized with a different random seed, and provided consistent results.
Randomization	For all methods, results for benchmarks and reannotation were calculated for 30 independent runs, each parameterized with a different random seed set from 0-29. For PCA calculation, data was shuffled with 30 different random seeds.
Blinding	No blinding was performed in the study because no animals or patients were treated in the study and computational algorithms were trained on preestablished datasets.

Reporting for specific materials, systems and methods

We require information from authors about some types of materials, experimental systems and methods used in many studies. Here, indicate whether each material, system or method listed is relevant to your study. If you are not sure if a list item applies to your research, read the appropriate section before selecting a response.

Materials & experimental systems

n/a	Involved in the study	
☒	☐	Antibodies
☒	☐	Eukaryotic cell lines
☒	☐	Palaeontology and archaeology
☒	☐	Animals and other organisms
☒	☐	Clinical data
☒	☐	Dual use research of concern

Methods

n/a	Involved in the study	
☒	☐	ChIP-seq
☒	☐	Flow cytometry
☒	☐	MRI-based neuroimaging
